# Low-Dose CT Image Denoising Based on Improved DD-Net and Local Filtered Mechanism

**DOI:** 10.1155/2022/2692301

**Published:** 2022-08-03

**Authors:** Hongen Liu, Xin Jin, Ling Liu, Xin Jin

**Affiliations:** ^1^School of Software, Yunnan University, Kunming 650091, Yunnan, China; ^2^Engineering Research Center of Cyberspace, Yunnan University, Kunming 650000, Yunnan, China

## Abstract

Low-dose CT (LDCT) images can reduce the radiation damage to the patients; however, the unavoidable information loss will influence the clinical diagnosis under low-dose conditions, such as noise, streak artifacts, and smooth details. LDCT image denoising is a significant topic in medical image processing to overcome the above deficits. This work proposes an improved DD-Net (DenseNet and deconvolution-based network) joint local filtered mechanism, the DD-Net is enhanced by introducing improved residual dense block to strengthen the feature representation ability, and the local filtered mechanism and gradient loss are also employed to effectively restore the subtle structures. First, the LDCT image is inputted into the network to obtain the denoised image. The original loss between the denoised image and normal-dose CT (NDCT) image is calculated, and the difference image between the NDCT image and the denoised image is obtained. Second, a mask image is generated by taking a threshold operation to the difference image, and the filtered LDCT and NDCT images are obtained by conducting an elementwise multiplication operation with LDCT and NDCT images using the mask image. Third, the filtered image is inputted into the network to obtain the filtered denoised image, and the correction loss is calculated. At last, the sum of original loss and correction loss of the improved DD-Net is used to optimize the network. Considering that it is insufficient to generate the edge information using the combination of mean square error (MSE) and multiscale structural similarity (MS-SSIM), we introduce the gradient loss that can calculate the loss of the high-frequency portion. The experimental results show that the proposed method can achieve better performance than conventional schemes and most neural networks. Our source code is made available at https://github.com/LHE-IT/Low-dose-CT-Image-Denoising/tree/main/Local Filtered Mechanism.

## 1. Introduction

Computed tomography (CT) is crucial in medical diagnosis and illness analysis [1–5]. As excessive CT scan probably causes a series of acute potential cancers and cases, the medical instrument usually adopts a CT radiation dose as low as possible to avert the damage to health. However, it is certain that reducing the radiation dose will cause information loss of human tissue, and a large amount of noise in the image may influence the accuracy of the diagnosis. Thus, how to reduce the noise in low-dose CT (LDCT) images and preserve the image information is one of the critical issues in medical image processing.

As reducing the dose of CT radiation can produce the projection data with a low signal-to-noise ratio, some methods utilize the nonlinear filter [6, 7] or the statistical characteristic of noise [8, 9] to reduce the noise in the projection data. In addition, some methods attempted to remove the noise and streak artifacts in LDCT images directly. To remove the streak artifacts fused in the tissue structure, approaches including nonlinear diffusion filter [10], sparse representation, and dictionary learning [11–13] were proposed. Due to the excellent performance, some natural image denoising algorithms [14, 15] were applied to remove the noise in LDCH images. These methods belong to postprocessing methods and aim to preserve the detailed information and remove the noise and artifacts simultaneously.

**Figure 1:**
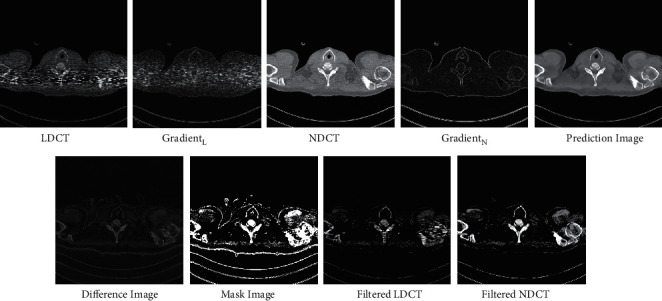
The comparison between the frequency-separation-based methods and local filtered mechanism. Gradient_*L*_ denotes the gradient image of the LDCT image. Gradient_*N*_ denotes the gradient image of the NDCT image. The gradient images were generated by the Sobel filter. The display window is [100, 300] Hu for better visualization.

Due to the remarkable expressive capacity of neural networks, many researchers try to reduce the noise in the LDCT images based on convolution neural network (CNN) and generative adversarial network (GAN). Some researchers utilized structures, such as autoencoder, residual block, and dense block, into CNN for LDCT image denoising [16–18]. Some researchers used CNN to learn image features and provide prior knowledge for traditional CT reconstruction schemes such as analytic reconstruction and iterative reconstruction [19, 20]. However, it is hard to generate realistic and diverse details based on CNN. To remedy this, some schemes based on GAN [21–28] were proposed. In these GAN-based solutions, the denoised images are generated by the generator and evaluated by the discriminator. Through competition and self-optimization, the generator can generate realistic normal-dose CT (NDCT) images. Besides, some novel structures, such as leap structure [29] and sharpness detector [23], were developed to enhance the performance of neural networks. Although these networks can perfectly remove the noise and recover the image structures, there are still some ambiguous and incorrect subtle structures in the denoised results. Confronting the problem, the frequency-separation-based networks [30, 31] were proposed to separate the LDCT image into a low-frequency portion and a high-frequency portion for generating each portion, respectively. However, the incorrect subtle structures are usually the areas that are contaminated severely by noise and streak artifacts instead of the edge information in high-frequency domain, as shown in Figure 1. On the one side, frequency separation usually produces information loss during transformation. On the other side, there is an essential correlation between global structures and detailed information, which is useful for subtle structure restoration.

To address the above problems, we use the difference image between the predicted image and the NDCT image to make the mask image. Compared with frequency-separation-based methods, the difference image can reflect the areas that contain incorrect subtle structures accurately. Using the mask image obtained by taking a threshold operation to the difference image, the local filtered mechanism filters the high-quality areas in the LDCT image and NDCT image and preserves the low-quality areas, which will be optimized by the network especially. To utilize the correlation between global structures and detailed information, our model learns the global structure between LDCT image and NDCT image in the first step. Then, the model learns the detailed information between the filtered LDCT image and NDCT image in the second step. By the network optimization in the second step, the ability in subtle structure restoration is enhanced significantly. As the global structures and detailed information are learned in the same network, our model can provide the implicit global features for subtle structure restoration and avoid the transformation loss during frequency separation. However, learning two tasks in one network requires a deeper network structure. Therefore, we propose an improved DD-Net [32] that has mighty ability in feature extraction and denoising. In our network, we replace the dense block by an improved residual dense block [33] to deepen the neural network and improve the performance. Although this mechanism solves the subtle structure restoration problem, the combination of MSE and MS-SSIM as a loss function is detrimental for the edge information restoration. Thus, we introduce the gradient loss [34] to calculate the loss in the high-frequency portion, which improves the performance reduction caused by the local filtered mechanism.

In this work, we evaluate the performance of our proposed network by comparing with other typical schemes. Experimental results demonstrate that the proposed scheme can restore the ambiguous subtle structures brilliantly and gets higher performance in objective metrics than most comparative methods. The contributions of the paper can be listed as follows:

- We propose an improved network based on DD-Net and a novel local filtered mechanism. Through this mechanism, the network can generate the subtle structures accurately with the global context and accomplish the balance between network generalization and subtle structure restoration.
- We introduce the gradient loss to enhance the ability in edge information restoration and improve the performance reduction caused by the abatement of generalization significantly.
- Experiments on low-dose chest image and brain image denoising prove that our network outperforms the conventional schemes and most neural networks in both evaluation metric and visual appearance.

## 2. Related Work

Since we propose an improved neural network for LDCT image denoising, some significant work will be reviewed optionally in this section.

As neural networks have achieved brilliant performance in image processing, LDCT denoising methods based on the neural network have been presented in the past decades. These methods achieved outstanding results in both objective metrics and visual appearance. Although analytic reconstruction and iterative reconstruction are still the mainstream in commerce scenes, it is certain that neural networks will be applied in commercial CT equipment in the future.

### 2.1. CNN Methods

Due to the powerful ability of feature extraction and mapping, some researchers attempted to reduce the noise in the LDCT images based on CNN. The deep residual network and cascade network [16–18] were the early applications to improve the performance of LDCT denoising. Zhang et al. [32] combined the dense block and deconvolution structure to build a lightweight network that can reuse the features effectively. Some methods [19, 20] combined neural network with analytic reconstruction or iterative reconstruction and improved the quality of LDCT images in the projection domain. Based on the residual block and dense block, the residual dense block (RDB) [33] achieved excellent performance in superresolution through contiguous memory (CM) mechanism and local feature fusion (LFF). By introducing the feature attention and enhancement attention modules (EAM), the real image denoising network (RIDNet) [35] can denoise real noise images efficiently. Inspired by the above study, this work introduces an improved residual dense block based on the DD-Net for achieving further enhancement of the feature representation and denoising.

### 2.2. GAN Methods

While methods based on CNN can greatly improve the denoising performance, they can only generate the image structures based on prior knowledge, which causes the restriction in subtle structure restoration. Therefore, the schemes based on GAN were presented. Yang et al. [21] applied the Wasserstein distance and perceptual loss to train the GAN network. Wolterink et al. [22] introduced the voxelwise loss to improve the performance of GAN. Ge et al. [24] developed a conditional GAN to generate the thin thickness slices from thick LDCH images. To compete with commerce algorithms, Shan et al. [25] proposed a modularized adaptive processing neural network (MAP-NN). Choi et al. [26] presented the semisupervised GAN, including denoising network and classification network, to reduce the dependence on NDCT images. Taking the consecutive low-dose projections as the input, the comprehensive domain generator [27] with three-dimension was presented to learn the redundant information among slices and generate the subtle structures. To capture structural details, You et al. [29] introduced the leap connection and network in network. To describe the uncertainty of the denoised image, Huang et al. [36] used the CutMix technique and U-Net-based discriminator to provide radiologists with a confidence map. However, although the GAN-based methods can perfectly preserve the texture information in LDCT images, they performed poorly in subtle structure restoration as well. In contrast, our network preserves the areas in low quality by the mask image and strengthens the ability in subtle structure restoration by optimizing the areas in low quality especially.

### 2.3. Subtle Structure Restoration

Currently, although CNN and GAN have remarkably improved the performance in image denoising, it is still hard to restore the subtle structures. Yin and Babyn [23] designed a sharpness detector based on cGAN to preserve more edge information. Wang et al. [30] applied the shearlet transformation to generate the high-frequency information and low-frequency information separately. Fritsche et al. [34] utilized the low pass filter for frequency separation and adopted the GAN loss for subtle structure restoration. Yang et al. [31] designed two subnetworks based on U-Net in the generator for LDCT image denoising in the spatial domain and high-frequency domain. Recent work restores subtle structures through frequency separation, which lacks global information and may bring information loss during transformation. In contrast, our network learns the global structures and detailed information in one network and can provide the implicit global context for subtle structure restoration. Meanwhile, our model can specially optimize the areas with low quality through the mask image and avoid information loss during transformation.

## 3. The Proposed Scheme

The critical content of LDCT image denoising is to restore the subtle structures while removing the noise. To enhance the ability in feature representation and denoising, we propose an improved DD-Net. Besides, we present a new local filter mechanism and introduce a novel gradient loss to restore subtle structures accurately.

### 3.1. Network Structure

**Figure 2:**
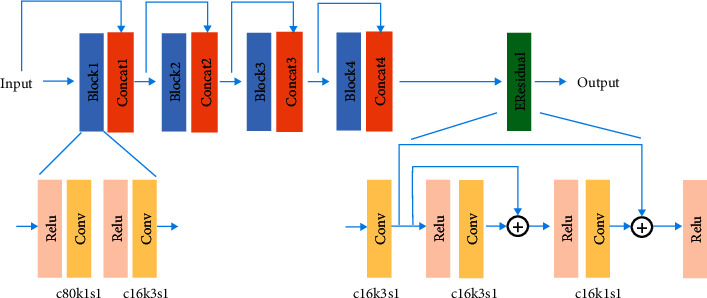
The structure of improved residual dense block (IRDB). c80k1s1 denotes that the channel, kernel size, and stride are 80, 1 × 1, and 1, respectively. c16k3s1 denotes that the channel, kernel size, and stride are 16, 3 × 3, and 1, respectively. c16k1s1 denotes that the channel, kernel size, and stride are 16, 1 × 1, and 1, respectively. EResidual denotes the enhanced residual block.

The proposed neural network employs a similar structure with DD-Net [32], which achieves brilliant performance in medical image denoising. However, as the global structures and detailed information are learned in one network, our model requires a deeper network structure and more powerful feature representation ability. Thus, the improved residual dense block (IRDB) [33] is introduced. The improved residual dense block is composed of the dense connected [37] block and the enhanced residual block [35]. Considering the scale variation in the max-pooling layers [38], we remove the batch normalization in the dense connected block. The detailed structure of the improved residual dense block is shown in Figure 2.

**Figure 3:**
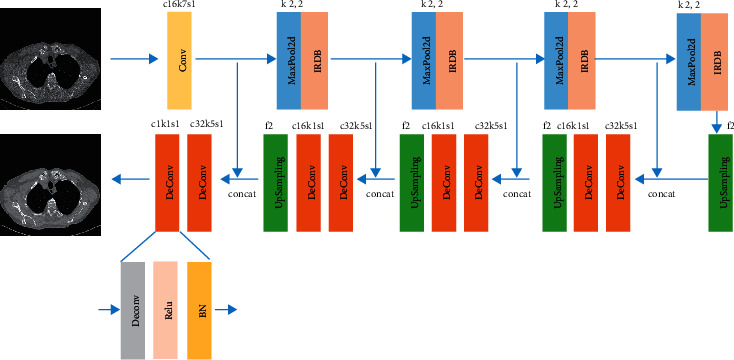
An overview of our network structure. The c16k7s1 denotes that the channel, kernel size, and stride are 16, 7 × 7, and 1, respectively. *k* 2, 2 denotes that the kernel size of max-pooling 2*d* is 2 × 2. *f*2 denotes that the upscale factor of upsampling is 2. c32k5s1 denotes that the channel, kernel size, and stride are 32, 5 × 5, and 1. c16k1s1 denotes that the channel, kernel size, and stride are 16, 1 × 1, and 1. c1k1s1 denotes that the channel, kernel size, and stride are 1, 1 × 1, and 1.

The detailed network structure is represented in Figure 3. It includes 1 convolution layer, 4 max-pooling layers, 4 improved residual dense blocks, 4 upsampling layers, and 8 deconvolution layers, followed by Relu and batch normalization.

The input and output of the network are 512 × 512 × 1 medical images. We adopt a 7 × 7 convolution layer after the input layer. After the 7 × 7 convolution layer, there are 4 encoder modules and 4 decoder modules. The encoder modules employ the max-pooling layer and the improved residual dense block [33] to extract multiscale features. The decoder modules employ the upsampling layers and 2 deconvolution layers followed by the Relu and batch normalization to restore the image information. The layers with the same feature shape in encoder modules and decoder modules will be concatenated. The kernel size of the last deconvolution layer is 1 × 1 to generate the denoised image.

### 3.2. Local Filtered Mechanism

**Figure 4:**
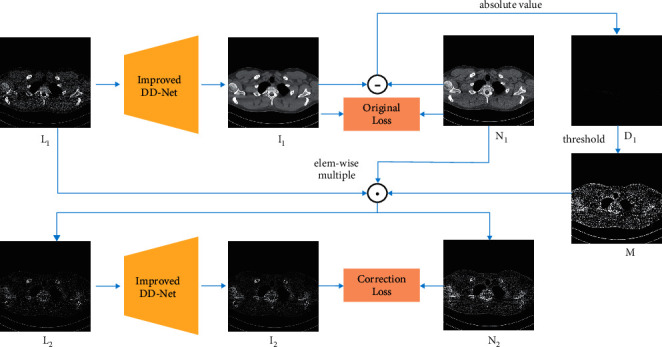
The framework of local filtered mechanism.

As the subtle structure restoration is a significant content in LDCT image denoising, most schemes attempt to restore subtle structure by frequency separation. However, high-frequency information cannot reflect the subtle structures accurately, and approaches that process high-frequency information and low-frequency information separately may cause information loss during the transformation. To solve the problem, we propose a local filter mechanism to enhance the ability in subtle structure restoration. The mechanism restores the unclear subtle structures by two steps. In the first step, the LDCT image is inputted into the network to get the denoised image *I*_1_. In the second step, the difference image *D*_1_ between the NDCT image and *I*_1_ is obtained. Then, the mask image is generated by taking the threshold operation to *D*_1_. Using the mask image to conduct elementwise multiplication operation with LDCT and NDCT images, the areas with high quality are filtered, and the areas with poor quality are preserved. At last, the filtered LDCT image is inputted into the network to get the filtered denoised image *I*_2_. The original loss between the denoised image *I*_1_ and NDCT image *N*_1_ is calculated to enhance the ability in global structure restoration. The correction loss between the filtered denoised images *I*_2_ and filtered NDCT image *N*_2_ is calculated to enhance the ability in unclear subtle structure restoration. The detailed process is shown in Figure 4 and Algorithm 1.

**Figure 5:**
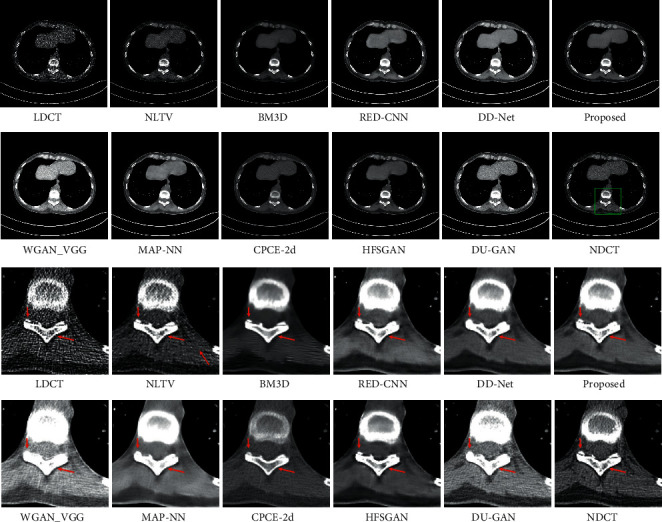
The denoised result for low-dose CT image in group 1. The loss function of DD-Net is the combination of MSE and MS-SSIM. The display window is [150, 250] Hu for better visualization.

**Figure 6:**
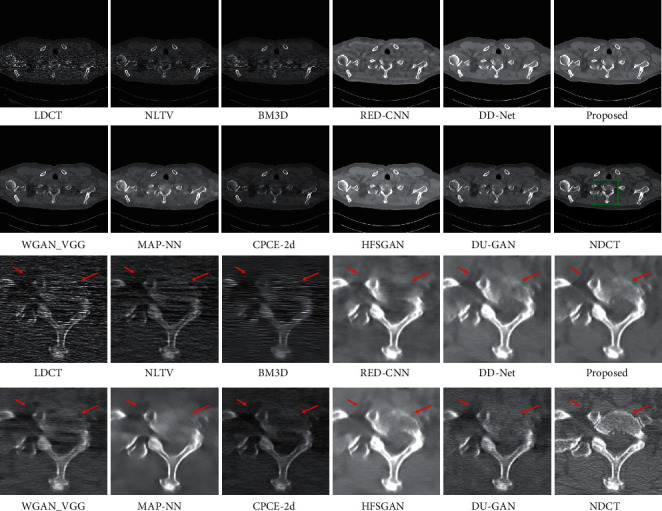
The denoised result for low-dose CT image in group 2. The display window is [100, 300] Hu for better visualization.

**Figure 7:**
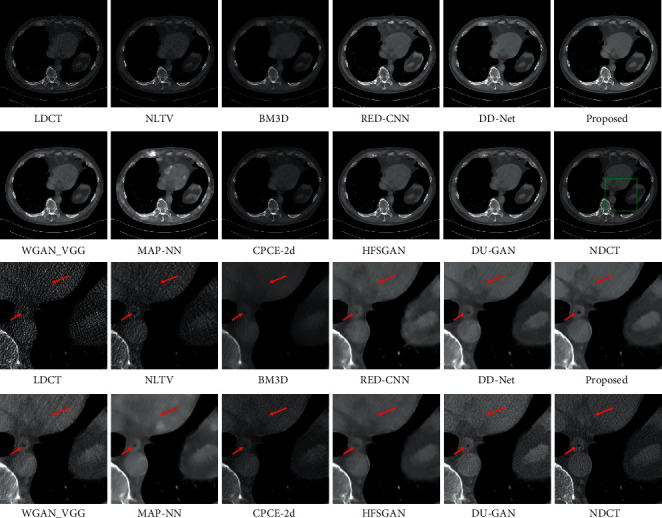
The denoised result for low-dose CT image in group 3. The display window is [100, 300] Hu for better visualization.

**Figure 8:**
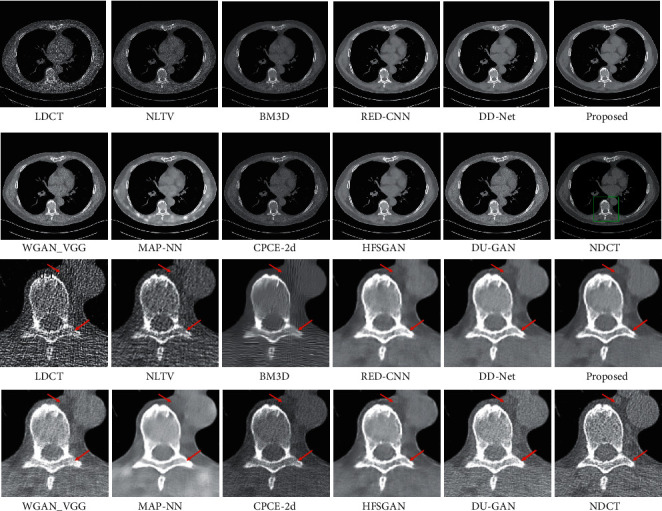
The denoised result for low-dose CT image in group 4. The display window is [100, 300] Hu for better visualization.

Compared with the frequency-separation-based methods, the proposed network can achieve feature sharing between global structure and detailed information and can provide more context information for detail restoration. Thus, it can generate more realistic and precise subtle structures, as shown in Figures 5–8. In fact, the above mechanism can be considered as a confrontation between network generalization and subtle structure restoration. The neural network tends to discard some detailed information of a specific image and make the subtle structures oversmoothed, which is beneficial for the enhancement of generalization and the reduction of overall error. To alleviate this phenomenon, this mechanism is designed to filter the areas with high quality and drive the network to specially optimize the areas with low quality that contain detailed information and are harder to optimize. Finally, the network can achieve the balance between the image denoising and subtle structure restoration through the sum of original loss and correction loss of the improved DD-Net.

**Figure 9:**
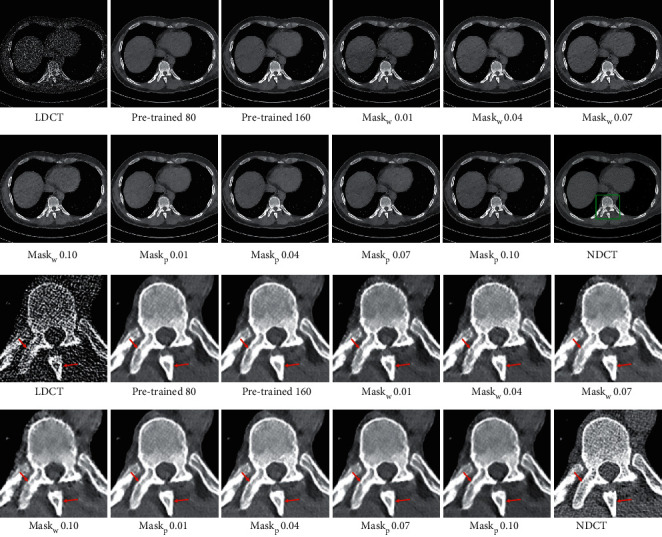
The denoised result for low-dose CT image with different threshold values and training strategies. The display window is [100, 300] Hu for better visualization. Pretrained 80 denotes the image generated by the model trained without local filtered mechanism for 80 epochs. Pretrained 160 denotes the image generated by the model trained without local filtered mechanism for 160 epochs. Both Mask_*w*_ and Mask_*p*_ denote the image generated by the model trained with local filtered mechanism. Mask_*w*_ denotes the image generated by the model trained without the pretrained model. Mask_*p*_ denotes the image generated by the model trained with the pretrained model.

Table 1 and Figure 9 demonstrate the results of different threshold values and training strategies. Although the model which was trained without a local filter mechanism can achieve higher PSNR and SSIM results, it drops some subtle structures to improve the generalization ability and cannot achieve further enhancement of subtle structure quality. The models which were trained with a local filter mechanism for 160 epochs can restore more appealing subtle structures but achieve poor performance in low-frequency portion denoising. Therefore, all networks without a pretrained model obtain lower PSNR and SSIM results than a pretrained model. The reason is that the global structures and detailed information are learned separately when adopting a local filtered mechanism at the beginning of the training phase. Without the prior knowledge of global structures, the network cannot utilize the global structure information learned in the first step to restore the subtle structures in the second step efficiently. Models with threshold values 0.01 and 0.04, which were trained with a pretrained model and local filter mechanism for 160 epochs, obtain higher PSNR and SSIM results than a pretrained model, but the improvement is slower than the model which was trained without a local filter mechanism for 160 epochs. The reason is that the subtle structures are more difficult to optimize. As is shown in Figure 9, it can be seen that all models trained with pretrained model and local filter mechanism pay more attention to subtle structure restoration.

**Figure 10:**
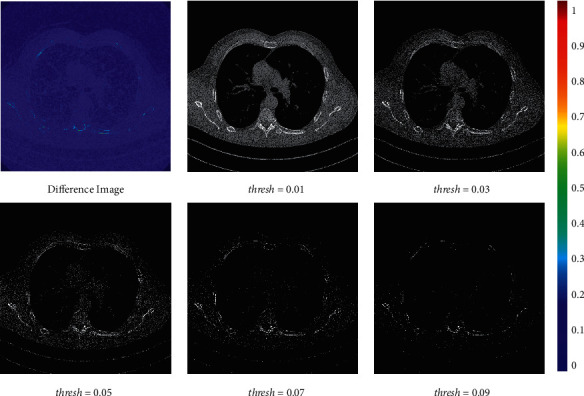
The difference image and filtered normal-dose CT images using different threshold values. The difference image was processed by pseudocolorization technology for better visualization. The display window is [0, 400] Hu for filtered normal-dose CT images.

Furthermore, how to set the threshold value is a crucial problem. When we use the low threshold value, the filtered images contain a large amount of low-frequency information which hinders the network from especially optimizing subtle structures. When we use the high threshold value, most subtle structures are filtered, and large black areas in the filtered images will influence the global structure restoration as there is a large difference between the filtered LDCT image and real LDCT image. Here, we trained the model without the local filtered mechanism for 80 epochs at first and visualized the filtered results using different threshold values. The visualization results are shown in Figure 10. As the threshold value of 0.04 can filter most low-frequency areas and save enough subtle structures, we choose 0.04 as our training threshold value in the chest dataset. Adopting the above parameter selection strategy, we choose 0.004 as our training threshold value in the brain dataset. Table 1 shows that both larger threshold value and lower threshold value will reduce the quality of the denoised results. The model whose threshold value is 0.04 obtains the highest PSNR and SSIM results in the chest dataset regardless of whether we use pretrained model or not. Furthermore, Figure 9 illustrates that an appropriate threshold can obtain a better visual appearance of subtle structures. Experiment verification indicates that the above parameter selection strategy can obtain a relatively proper threshold value.

### 3.3. Loss Functions

In our experiments, we use the weighted sum of mean square error, multiscale structural similarity [39], and gradient loss as the final loss function. To implement the local filter mechanism, it is required to calculate the original loss between the denoised image *I*_1_ and NDCT image *N*_1_ when the LDCT image *L*_1_ is used as the input, as well as the correction loss between the denoised image *I*_2_ and filtered NDCT image *N*_2_ when the filtered LDCT image *L*_2_ is used as the input. Thus, the total loss function is the sum of original loss and correction loss of the improved DD-Net, as equations (1) and (2).

(1) $$\begin{array}{c} L=\lambda_{1}L_{MSE}+\lambda_{2}L_{MS-SSIM}+\lambda_{3}L_{gra\;d}. \end{array}$$

(2) $$\begin{array}{c} L_{\text{total }}=L_{\text{original }}+L_{\text{correct }}, \end{array}$$

where *λ*_1_, *λ*_2_, and *λ*_3_ are the hyperparameters.

#### 3.3.1. Mean Square Error (MSE)

The mean square error (MSE) is used to generate the objective information of images and calculate the pixel difference between denoised images and NDCT images. The MSE is described in equations (3) and (4).

(3) $$\begin{array}{c} I\left(i,j\right)=\text{ImprovedDDNet}\left(L\left(i,j\right)\right). \end{array}$$

(4) $$\begin{array}{c} L_{MSE}=\frac{1}{N^{2}}\sum_{i=1}^{N}\sum_{j=1}^{N}{\left(N\left(i,j\right)-I\left(i,j\right)\right)}^{2}, \end{array}$$

where *I*(*i*, *j*) denotes the gray value of denoised images at pixel (*x*, *y*) and *N*(*i*, *j*) denotes the gray value of NDCT images at pixel (*x*, *y*).

#### 3.3.2. Multiscale Structural Similarity (MS-SSIM)

The structural similarity (SSIM) is a common metric for evaluating the perceptual loss in a single scale. As optimization of SSIM, the multiscale structural similarity (MS-SSIM) [39] is conducted over different resolutions and has a better performance. The MS-SSIM is described in equations (5)–(9).

(5) $$\begin{array}{c} l\left(x,y\right)=\frac{2\mu_{x}\mu_{y}+C_{1}}{\mu_{x}^{2}+\mu_{y}^{2}+C_{1}}\\ s\left(x,y\right)=\frac{\sigma_{xy}+C_{3}}{\sigma_{x}\sigma_{y}}. \end{array}$$

(6) $$\begin{array}{c} \text{MS}-\text{SSIM}\left(x,y\right)={\left[l_{M}\left(x,y\right)\right]}^{\alpha_{M}}\cdot \prod_{j=1}^{M}{\left[c_{j}\left(x,y\right)\right]}^{\beta_{j}}\cdot {\left[s_{j}\left(x,y\right)\right]}^{\gamma_{j}}. \end{array}$$

(7) $$\begin{array}{c} L_{\text{MS}-\text{SSIM}}=1-\text{MS}-\text{SSIM}. \end{array}$$

(8) $$\begin{array}{c} \alpha_{j}=\beta_{j}=\gamma_{j}. \end{array}$$

(9) $$\begin{array}{c} \sum_{j=1}^{M}\gamma_{j}=1, \end{array}$$

where *I*(*x*, *y*) is used to evaluate luminance, *c*(*x*, *y*) is used to evaluate the contrast, and *s*(*x*, *y*) is used to evaluate the structural similarity. *α*, *β*, *γ* are constants. In general, *α*_1_ = *β*_1_ = 0.0448, *α*_2_ = *β*_2_ = 0.2856, *α*_3_ = *β*_3_ = 0.3001, *α*_4_ = *β*_4_ =  0.2363, and *α*_5_ = *β*_5_ = 0.1333. *C*_1_, *C*_2_, *C*_3_ are constants. *C*_1_=(0.01 · (2^*B*^ − 1))^2^, *C*_2_=(0.03 · (2^*B*^ − 1))^2^, *C*_3_=(*C*_2_/2), and *B* denotes the bit depth of the image.

#### 3.3.3. Gradient Loss

The gradient loss [34] is used to generate the edge information of images. In the first step, we take the convolution operation with denoised image and NDCT image, respectively, using a high-pass filter kernel *K* instead of the Sobel operator. In the second step, the gradient loss can be obtained by calculating the mean square error between the denoised image *G*_predict_ and NDCT image *G*_normal_. The gradient loss is described in equations (10)–(13).

(10) $$\begin{array}{c} K=\left[\begin{array}{ccc} -1 & -1 & -1\\ 0 & 0 & 0\\ 1 & 1 & 1 \end{array}\right]. \end{array}$$

(11) $$\begin{array}{c} G_{\text{predict}}=I⊗K. \end{array}$$

(12) $$\begin{array}{c} G_{\text{normal }}=H⊗K. \end{array}$$

(13) $$\begin{array}{c} L_{\text{grad }}=\mathrm{MSE}\left(G_{\text{predict }},G_{\text{normal }}\right), \end{array}$$

where *K* denotes the high-pass filter kernel and ⊗ denotes the convolution operation.

## 4. Experiments

The experimental details and result analysis are introduced in this section to validate the effectiveness of our proposed scheme.

### 4.1. Dataset and Implement Details

#### 4.1.1. Dataset

In our experiments, the dataset comes from the “2016 NIH-AAPM Mayo Clinic Low Dose CT Grand Challenge.” It comprises 100 chest scans with 10% of the routine dose, 99 head scans with 25% of the routine dose, and 100 abdomen scans with 25% of the routine dose. In our study, 10% chest scan and 25% head scan are used as the chest dataset and brain dataset. For chest dataset, we randomly select 25 cases for training and 10 cases for testing. For brain dataset, we randomly select 40 cases for training and 10 cases for testing. There is no overlap between training and testing. The medical image dataset can be obtained from the Cancer Imaging Archive (TCIA) website at https://wiki.cancerimagingarchive.net/pages/viewpage.action?pageId=52758026.

#### 4.1.2. Details

**Figure 11:**
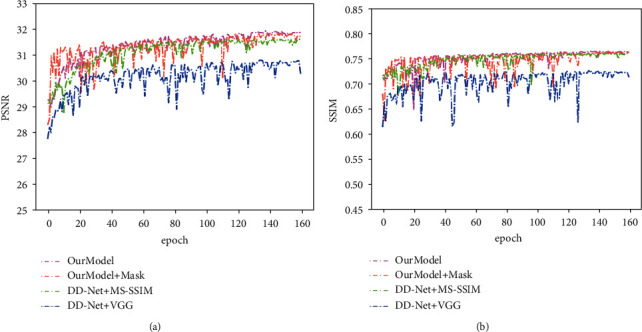
The curves of PSNR and SSIM on the chest testing dataset during the network training.

**Figure 12:**
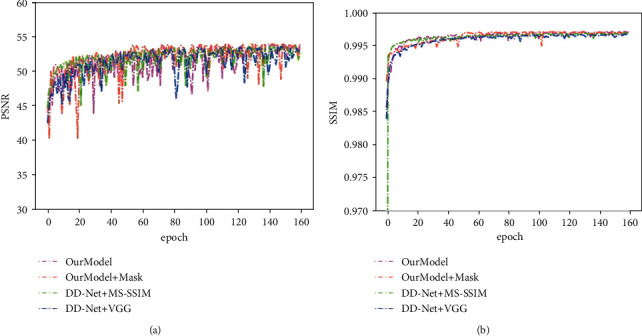
The curves of PSNR and SSIM on the brain testing dataset during the network training.

We trained and tested the proposed model in the chest dataset and brain dataset, respectively. The network was trained for 80 epochs without local filtered mechanism to obtain the pretrained model. Based on the pretrained model, the model was trained for 160 epochs with a local filtered mechanism to obtain the final model. The batch size was 8. The optimizer was Adam with *β*_1_ as 0.9 and *β*_2_ as 0.999. The initial learning rate was set to 1 × 10^−4^ which reduced to 5 × 10^−5^ at the 130-th epoch. The convolution and deconvolution layers were initialized with the Gaussian function whose mean square was 0, and variance was 0.01. We, respectively, set *λ*_1_, *λ*_2_, and *λ*_3_ to 1, 0.15, and 0.8 as the hyperparameters of loss function. All models were trained and tested on the NVIDIA RTX 2080 Ti GPU. Besides, the training curves in Figures 11 and 12 demonstrate that our method is easier to achieve higher performance than DD-Net using MS-SSIM. Although the model which was trained without local filtered mechanism can get higher PSNR and SSIM results, it will drop some subtle structures to improve the generalization ability, which is shown in Figure 9. The PSNR and SSIM results on the chest testing dataset, respectively, stabilize at 31.80 and 0.76 when the epoch is 120. The PSNR and SSIM results on the brain dataset, respectively, stabilize at 53.70 and 0.99 when the epoch is 140.

To verify the effectiveness of our scheme in subtle structure restoration, we selected some competitive methods for evaluation. Nonlocal total variation (NLTV) [40] is known as a statistical iterative method based on the compressed sensing (CS) technique. It adopts the global search and nonuniform weight penalization to improve the denoised image quality. Block-matching and 3d filtering (BM3D) [15] is the postprocessing method. It has the advantages of nonlocal methods and transform methods and removes the noise by searching and matching the similar blocks. Residual encoder-decoder convolution neural network (RED-CNN) [16] is a CNN-based method. It utilizes the residual structure and encoder-decoder structure and takes MSE as a loss function. Different from RED-CNN, DD-Net [32] applies the dense block in the encoder-decoder structure and uses the combination of MSE and SSIM as a loss function. Generative adversarial network with Wasserstein distance and perceptual loss (WGAN-VGG) [21] is a GAN-based method. It takes the combination of perceptual similarity calculated by the VGG-19 network and Wasserstein distance as a loss function. Adopting the GAN structure as well, MAP-NN [25] applies the multiple conveying path-based convolution encoder-decoder (CPCE) modules in the generator. It takes the combination of MSE, Wasserstein distance, and edge incoherence calculated by the Sobel operator as a loss function. 2D conveying path-based convolution encoder-decoder network (CPCE-2d) [28] uses single CPCE module for LDCT denoising and takes the combination of adversarial loss and perceptual loss as the loss function. High-frequency sensitive generative adversarial network (HFSGAN) [31] is a frequency-separation-based method. It obtains high-frequency portion and low-frequency portion by guided filter and applies two U-Net to process the high-frequency portion and whole image separately. Generative adversarial networks with dual-domain U-Net-based discriminators (DU-GAN) [36] apply two U-Net-based discriminators to evaluate the difference in image domain and gradient domain and adopt the CutMix technique to describe the uncertainty of the denoised image.

In the NLTV method, we set the number of iterations, time step (d*t*), gradient regularization (*ϵ*), and fidelity term (*λ*) to 5, 0.2, 1 × 10^−6^, and 1.2, respectively. In the BM3D method, the parameter *σ* was 25, and the hard threshold value was 2.7 × *σ*. *β* in Kaiser filter was 2. In the basic estimation, we set the match threshold value, maximum number of group matched blocks, block size, block stride, search step, and search window size to 2500, 16, 8, 3, 3, and 39, respectively. In the final estimation, we set the match threshold value, maximum number of group matched blocks, block size, block stride, search step, and search window size to 400, 32, 8, 3, 3, and 39, respectively. In the RED-CNN method, the LDCT images were cropped into 64 × 64 patches as the input, and the batch size was 160. The network was trained for 100 epochs using Adam with default parameters; namely, *β*_1_ was 0.9 and *β*_2_ was 0.999. The initial learning rate was set to 1 × 10^−5^ and decreased by 0.5 every 30 epochs. In the DD-Net method, it took the 512 × 512 images as the input, and the batch size was 8. The network was trained for 160 epochs using Adam with default parameters. The initial learning rate was set to 1 × 10^−4^ and slowly decreased to 1 × 10^−5^. In the WGAN-VGG method, the LDCT images were cropped into 64 × 64 patches as the input, and the batch size was 128. The network was trained for 200 k iterations using Adam with *β*_1_ as 0.5 and *β*_2_ as 0.9, respectively. The learning rate was set to 1 × 10^−5^, and *λ*_1_ in the loss function was set to 0.1. *λ*_*p*_ for the gradient penalty was 10. In the MAP-NN method, the LDCT images were cropped into 64 × 64 patches as the inputs, and the batch size was 128. The network was trained for 80 epochs using Adam with default parameters. The initial learning rate was set to 1 × 10^−4^ and decreased by $1/\sqrt{t}$ after the *t*-th epoch. *λ*_*m*_ and *λ*_*e*_ in the loss function were set to 50 and 50, respectively. *λ*_*p*_ for the gradient penalty was 10. The number of conveying-link-oriented network encoder-decoders (CLONE) was 5. In the CPCE-2d method, the LDCT images were cropped into 64 × 64 patches as the inputs, and the batch size was 128. The network was trained for 40 epochs using Adam with default parameters. The initial learning rate was set to 1 × 10^−4^ and decreased by 1/*t* after the *t*-th epoch. *λ*_*p*_ for perceptual loss was set to 0.1. In the HFSGAN method, it took the 512 × 512 images as the input, and the batch size was 12. The network was trained for 200 epochs using Adam with *β*_1_ as 0.5 and *β*_2_ as 0.999, respectively. The initial learning rate was set to 2 × 10^−4^. *λ*_1_ and *λ*_2_ in the loss function were set to 100 and 50, respectively. In the DU-GAN method, the LDCT images were cropped into 64 × 64 patches as the inputs, and the batch size was 64. The network was trained for 100000 iterations using Adam with default parameters. The initial learning rate was 1 × 10^−4^. *λ*_adv_, *λ*_img_, and *λ*_grd_ in the loss function were set to 0.1, 1, and 20, respectively. The code of NLTV was downloaded at http://math.sjtu.edu.cn/faculty/xqzhang/NLIP_v1.zip. The code of BM3D was implemented using Python. Other deep learning-based methods were implemented using PyTorch according to the official codes.

### 4.2. Evaluation Metrics

Appropriate evaluation metrics are crucial for the evaluation of LDCT image denoising because medical images contain more subtle structures and fewer channels than natural images. We select the peak signal-to-noise ratio (PSNR) and structural similarity (SSIM) as the evaluation metrics. These evaluation metrics are widely used in image processing tasks, such as image superresolution and image inpainting.

#### 4.2.1. Peak Signal-to-Noise Ratio (PSNR)

PSNR is an objective metric to measure the error of image pixels, and it is usually used in the images which are sensitive to error, as equations (14)-(15). In general, the higher the PSNR is, the lower distortion the image has.

(14) $$\begin{array}{c} MSE=\frac{1}{N^{2}}\sum_{i=1}^{N}\sum_{j=1}^{N}{\left(x\left(i,j\right)-y\left(i,j\right)\right)}^{2}. \end{array}$$

(15) $$\begin{array}{c} PSNR=10\cdot \;\log_{10}\left(\frac{MAX^{2}}{MSE}\right), \end{array}$$

where *x*, *y*, *N* denote the denoised image, normal-dose image, and the width or height of the image, respectively, and MAX denotes the maximum of gray value.

#### 4.2.2. Structural Similarity (SSIM)

As the PSNR cannot completely reflect the subjective visual difference, the SSIM is used as a supplement to measure the visual appearance of images, as equation (16). In general, the higher the SSIM is, the more abundant and appealing visual appearance the image has.

(16) $$\begin{array}{c} MS-SSIM=\frac{\left(2\mu_{x}\mu_{y}+c_{1}\right)\left(2\sigma_{xy}+c_{2}\right)}{\left(\mu_{x}^{2}+\mu_{y}^{2}+c_{1}\right)\left(\sigma_{x}+\sigma_{y}+c_{2}\right)}, \end{array}$$

where *μ* denotes the mean value, *σ* denotes the variance, *σ*_*xy*_ denotes the covariance of the denoised image and normal-dose image, and *c*_1_ and *c*_2_ are constants. *c*_1_=(0.01 · MAX)^2^, and *c*_2_=(0.03 · MAX)^2^.

### 4.3. Experimental Results and Analysis

#### 4.3.1. Qualitative Evaluations

The LDCT denoised images are shown in Figures 5–8. The regions of interest (ROIs) are marked by green rectangles, and the red pointing arrows denote the difference between our method and comparative methods.

From the enlarged view under the denoised results, it can be seen that the BM3D and NLTV reduce the noise to some extent but fail to remove the artifacts, which is shown in Figure 6. The denoised images generated by RED-CNN and MAP-NN are slightly oversmoothed due to adopting MSE as a loss function. As the perceptual loss and adversarial loss cannot reflect the pixel level difference accurately, the WGAN-VGG and CPCE-2d preserve the texture information in LDCT images but cannot remove the streak artifacts completely. Using the MS-SSIM as a loss function, the DD-Net can remove the noise and streak artifacts effectively but cannot restore some subtle structures accurately, which can be shown in Figure 7. As the frequency-separation-based method, the HFSGAN enhanced the structural similarity between the denoised image and NDCT image in high-frequency portion and can restore the subtle structures clearly. However, it blurred the image information in the low-frequency portion. As is shown in Figure 6, the DU-GAN can generate the image with realistic texture information but cannot restore the subtle structure when the LDCT image is contaminated severely by noise and streak artifacts. Overall, as is shown in Figures 6 and 7, our method can restore subtle structures effectively and keep the color and structure consistent with the NDCT image.

#### 4.3.2. Quantitative Evaluations

To validate the performance of our neural network quantitatively, we adopted peak signal-to-noise ratio (PSNR) and structural similarity (SSIM) as objective metrics.  Table 2 shows the PSNR and SSIM results of the chest dataset and brain dataset using different methods. The NLTV and BM3D are traditional denoising methods, the RED-CNN, DD-Net, and our model are CNN-based denoising methods, and the WGAN-VGG, MAP-NN, CPCE-2D, HFSGAN, and DU-GAN are GAN-based denoising methods.

First, as the NLTV and BM3D remove the noise and artifacts through the information of single LDCT image solely, they perform poorly in image information restoration and get the lower PSNR and SSIM results than deep learning-based methods. Second, as the perceptual loss and adversarial loss reflect the style difference, the WGAN-VGG and CPCE-2d cannot remove the streak artifacts completely. Therefore, they obtain lower PSNR and SSIM results than methods using MSE as one of the loss functions, including RED-CNN, DD-Net, MAP-NN, HFSGAN, DU-GAN, and our method in the chest dataset. Meanwhile, as the RED-CNN was trained with MSE loss solely, it produces oversmoothed denoised results in Figure 6. Due to the MS-SSIM loss, the denoised results generated by DD-Net keep the high structural similarity with NDCT images. Therefore, the DD-Net obtains the second-best PSNR and SSIM results in the chest dataset and the third-best PSNR and SSIM results in the brain dataset. As the HFSGAN processes the high-frequency portion especially, it can accurately restore the subtle structures. However, it blurs the image information in the low-frequency portion and obtains lower objective metrics results than DD-Net and our model in the chest dataset. In addition, due to the information loss during frequency separation, the HFSGAN performs poorly when the difference between LDCT images and NDCT images is relatively small. Therefore, the HFSGAN obtains the lowest PSNR and SSIM results among deep learning-based methods in the brain dataset. Although the DU-GAN can preserve the texture information in LDCT images, it performs poorly in subtle structure restoration, which can be shown in Figure 6, and cannot keep color consistency with NDCT images. Besides, as there is a relatively large difference in texture information between LDCT images and NDCT images, the DU-GAN obtains lower PSNR and SSIM results than most deep learning-based methods in the chest dataset. However, when the difference between LDCT images and NDCT images is small, the DU-GAN can accurately sense the difference through a confidence map and get the best PSNR result in the brain dataset. Our model achieves high performance in subtle structure restoration and obtains the best objective metrics results in the chest dataset. However, our model obtains the second-best PSNR result and the best SSIM result in the brain dataset because the confidence map and taking the 64 × 64 patches as the input in the DU-GAN can sense the difference with low confidence score more accurately than our method. In contrast, our model can achieve higher performance when the LDCT images contain a large amount of noise and streak artifacts.

**Figure 13:**
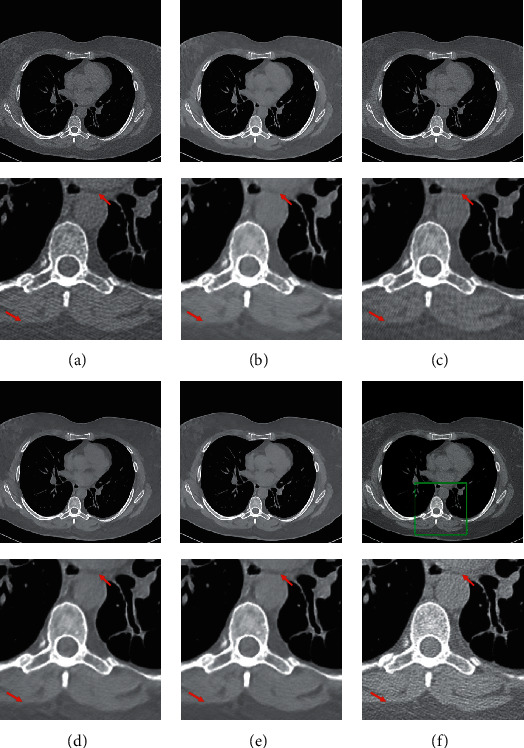
The denoised results generated by different loss functions and neural networks. (a) DD-Net + VGG. (b) DD-Net + MS-SSIM. (c) Proposed + VGG. (d) Proposed + MS-SSIM. (e) Proposed + MS-SSIM + local filtered mechanism + gradient loss. (f) NDCT. The display window is [100, 300] for better visualization.

Because the perceptual loss using VGG-19 [41] is another effective loss function to enhance the visual appearance, it is compared with MS-SSIM. As is shown in Figures 11 and 12, the model using MS-SSIM as a loss function obtains higher PSNR and SSIM results than perceptual loss using VGG-19. The reason is that VGG-19 is trained on natural images and cannot reflect the visual features of medical images perfectly. Moreover, as shown in Figure 13, whether it is DD-Net or our network, the image generated by the model training with VGG-19 contains more noise than the model using MS-SSIM.

#### 4.3.3. Uncertainty Visualization

**Figure 14:**
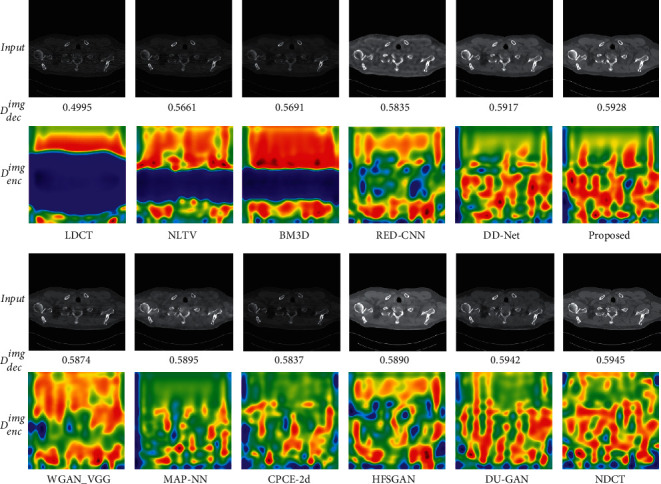
Uncertainty visualization of applying the trained discriminator to the outputs of different methods. The display window is [100, 300] Hu for better visualization. Note that the blue color of *D*_dec_^img^ indicates a lower confidence score while the red color indicates a high confidence score.

The uncertainty visualization of different methods is shown in Figure 14. As both BM3D and NLTV reduce the noise in the LDCT image to some extent, they can obtain higher global scores of *D*_dec_^img^ than the LDCT image. However, they cannot remove the streak artifacts and restore the image information efficiently, which causes low per-pixel confidence. The RED-CNN can efficiently remove the noise and streak artifacts but oversmoothens the LDCT image simultaneously. Therefore, its global score and per-pixel confidence are lower than other deep learning-based methods. In addition, although the WGAN-VGG and CPCE-2d can avoid the oversmooth problem, they cannot remove streak artifacts completely. Therefore, their global scores are higher than RED-CNN but lower than most deep learning-based methods. The MAP-NN will pay more attention to subtle structure restoration by adopting the gradient loss as the loss function but blurs the low-frequency portion according to per-pixel confidence. Therefore, it obtains a higher global score and per-pixel confidence than WGAN-VGG. The HFSGAN can generate realistic subtle structures but oversmoothens the low-frequency portion in the LDCT image as well according to the confidence map. The DD-Net can remove the noise and streak artifacts efficiently and keep high structural similarity with the NDCT image according to the confidence map. However, compared with our method, it cannot restore some subtle structures accurately and obtains a lower global score than our method. As the DU-GAN is trained with the discriminator and can adjust the image quality pertinently, it can generate the photo-realistic denoised results according to the confidence map and obtains the best global score. The proposed method obtains higher per-pixel confidence than DU-GAN in the subtle structures and obtains the second-best global score, indicating that our method achieves better performance in subtle structure restoration.

### 4.4. Ablation Study

Since we introduce some novel structures and local filtered mechanism based on DD-Net, it is significant to take a comparative analysis. The detailed results are presented in Table 3, which shows the objective metrics of our modifications.

#### 4.4.1. Use Improved Residual Dense Block

As the global structure and detailed information are learned in one network, we introduce the improved residual dense block to improve the representation ability of the neural network. As is shown in Table 3, our model obtains higher PSNR and SSIM results than DD-Net when the network uses the improved residual dense block and does not introduce other improvements. Furthermore, when the DD-Net was trained with local filtered mechanism, using the improved residual dense block can solve the performance reduction remarkably, indicating that the representation ability of DD-Net is insufficient for the local filtered mechanism, and introducing the above module can enhance the performance in subtle structure restoration.

#### 4.4.2. Use Local Filtered Mechanism

As the high-frequency portion cannot reflect the incorrect subtle structures accurately and frequency-separation-based methods cannot utilize the correlation between global structures and detailed information, we introduce the local filtered mechanism. Filtering the areas with high quality, this mechanism drives the network to specially optimize the subtle structures in low quality and enhances the ability in subtle structure restoration. However, it causes performance reduction but can make the network generate more precise and realistic subtle structures. The reason is that the areas preserved by this mechanism are more difficult to optimize, and this mechanism hinders the network from dropping some subtle structures for better generalization ability.

#### 4.4.3. Use Gradient Loss

As the combination of MSE and SSIM cannot keep the edge information consistency between the denoised result and NDCT image, the network will oversmoothen some subtle structures, which is shown in Figure 7. To remedy this, we introduce the gradient loss to sense the edge information in the LDCT image. As is shown in Table 3, introducing the gradient loss can solve the performance reduction caused by the local filtered mechanism efficiently as well. In addition, as is shown in Figure 13, the color and edge information of subtle structures are enhanced through the local filtered mechanism and gradient loss, indicating that introducing the gradient loss is beneficial for subtle structure restoration.

## 5. Conclusion

In this work, we propose a novel scheme for LDCT denoising based on improved DD-Net and local filtered mechanism. As the incorrect subtle structures are usually the areas that are contaminated severely by noise and streak artifacts instead of the edge information in high-frequency domain, previous studies cannot restore the subtle structures efficiently. Therefore, we present the local filtered mechanism to filter the areas with high quality and make the network optimize the subtle structures especially. Based on the original loss and correction loss of the improved DD-Net, the proposed method can accomplish the balance between network generalization and subtle structure restoration. However, as learning global structures and detailed information in one network requires more powerful feature representation ability and the edge information is significant for subtle structure restoration, we introduce the improved residual dense block and gradient loss to deepen the network structures and keep the edge information consistency between the denoised result and NDCT image, respectively. The ablation study validates the effectiveness of the above components.

The quantitative results show that our scheme can obtain higher scores in objective metrics than conventional schemes and most neural networks. Meanwhile, the visual comparison and uncertainty visualization also show that our scheme can provide a brilliant approach for subtle structure restoration in LDCT image denoising. In addition, the proposed network achieves competitive performance in both chest dataset and brain dataset, even if their radiation doses are quite different, which demonstrates the generalization ability of our network in different scenarios. However, training the network needs to, respectively, calculate the original loss and correction loss of the improved DD-Net for each batch, which introduces more computational cost. Moreover, the effectiveness of our scheme requests further validation in other image processing tasks, such as image superresolution and restoration, which is a significant research direction in the future.

## Figures and Tables

**Algorithm 1:**
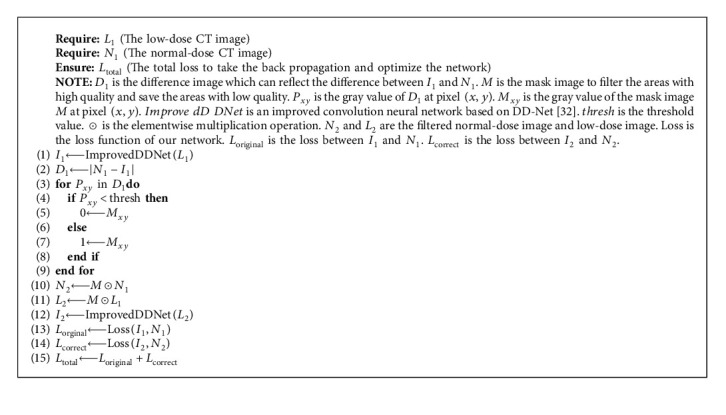
The local filtered mechanism.

**Table 1:** The PSNR and SSIM results of chest datasets using different threshold values and training strategies. The w/o mask denotes that the model was trained without local filter mechanism. The w/o pretrained 80 denotes that the model was trained with local filter mechanism for 160 epochs. Pretrained 80 denotes that the model was trained without local filtered mechanism for 80 epochs at first and was trained with local filter mechanism for 160 epochs.

Epoch	Threshold	Method	PSNR	SSIM
80	—	W/o mask	31.7071	0.7601
160	—	W/o mask	31.9231	0.7636
160	0.01	W/o pretrained 80	30.9619	0.7423
160	0.04	W/o pretrained 80	31.3512	0.7511
160	0.07	W/o pretrained 80	31.1152	0.7464
160	0.10	W/o pretrained 80	30.5216	0.7367
160	0.01	Pretrained 80	31.8175	0.7614
160	0.04	Pretrained 80	31.8401	0.7613
160	0.07	Pretrained 80	31.6834	0.7589
160	0.10	Pretrained 80	31.5151	0.7583

**Table 2:** The PSNR and SSIM results of chest dataset and brain dataset using different methods. The chest-10% denotes that the dose of low-dose chest images is 10% of the routine dose. The brain-25% denotes that the dose of low-dose brain images is 25% of the routine dose.

Method	Chest-10%		Brain-25%	
	PSNR	SSIM	PSNR	SSIM
LDCT	23.1989	0.4487	51.4490	0.9938
NLTV [40]	25.6894	0.6236	35.1604	0.8905
BM3D [15]	26.9452	0.6748	40.9885	0.7938

RED-CNN [16]	30.9656	0.7518	53.1678	0.9961
DD-Net + MS-SSIM [32]	31.6241	0.7595	53.6189	0.9967
DD-Net + VGG	30.8235	0.7240	53.4942	0.9966
Proposed	31.8401	0.7613	53.8237	0.9970

WGAN-VGG [21]	29.2275	0.6932	48.0044	0.9897
MAP-NN [25]	30.6552	0.7567	52.0912	0.9952
CPCE-2d [28]	27.5885	0.6889	50.7368	0.9935
HFSGAN [31]	31.0291	0.7468	38.5513	0.9162
DU-GAN [36]	29.9207	0.7012	54.0428	0.9968

**Table 3:** The ablation study in the chest dataset of our experiments.

Improved residual dense block [33]	Local filtered mechanism	Gradient loss [34]	PSNR	SSIM
×	×	×	31.6241	0.7595
✓	×	×	31.7742	0.7618
×	✓	×	31.6130	0.7569
×	×	✓	31.8317	0.7593
×	✓	✓	31.7400	0.7571
✓	✓	×	31.7363	0.7622
✓	×	✓	31.9231	0.7636
✓	✓	✓	31.8401	0.7613

## Data Availability

The data supporting this work are from previously reported studies and datasets, which have been cited. The processed data are available at https://wiki.cancerimagingarchive.net/pages/viewpage.action?pageId=52758026.
